# Research progress on cellular senescence in the pathogenesis and treatment of osteoarthritis and temporomandibular joint osteoarthritis

**DOI:** 10.22514/jofph.2026.033

**Published:** 2026-05-12

**Authors:** Yuan Wang, Xiaohui Jing, Yu Li

**Affiliations:** ^1^Department of Stomatology, Xuanwu Hospital, Capital Medical University, 100053 Beijing, China; ^2^Department of Stomatology, Beijing Stomatological Hospital, Affiliated to Capital Medical University, 100070 Beijing, China; ^3^Obstetrics and Gynecology, Pingliang Traditional Chinese Medicine Hospital, 744000 Pingliang, Gansu, China; ^4^Department of General Surgery, Xuanwu Hospital, Capital Medical University, 100053 Beijing, China

**Keywords:** Cellular senescence, Osteoarthritis, Temporomandibular joint arthritis, Anti-senescence drugs

## Abstract

Osteoarthritis (OA) is a common joint disorder characterized primarily by cartilage degeneration and osteophyte formation, leading to a substantial decline in patients’ quality of life. Temporomandibular joint OA (TMJOA) is a degenerative lesion within temporomandibular joint disorders, accounting for approximately 8%–16% of diagnosed cases. Its clinical manifestations include joint pain, limited mouth opening, joint noises, and related symptoms. Cellular senescence plays a pivotal role in OA pathogenesis. Senescent processes contribute to functional impairment of chondrocytes, synovial cells, and osteocytes through multiple signaling pathways. DNA damage, telomere attrition, oxidative stress, and the release of inflammatory mediators are major drivers of cellular senescence. However, current literature lacks a systematic integration of senescence-related mechanisms in OA and TMJOA. Furthermore, anti-aging therapeutic strategies for these conditions lack targeted approaches that account for interactions among distinct senescence mechanisms. This review elucidates the various characteristic types of cellular senescence, their interactions, and the senescence-induced pathogenesis of OA and TMJOA. A comprehensive investigation into the mechanisms of cellular senescence may yield novel insights and inform the development of therapeutic strategies for managing OA.

## 1. Introduction

Osteoarthritis (OA) is the most common joint disorder, characterized by degeneration and destruction of articular cartilage, synovial inflammation, subchondral bone remodeling, osteophyte formation along joint margins, and thickening of the joint capsule, ultimately leading to pain and functional impairment [1, 2]. Driven by population aging, increasing obesity rates, and rising incidence of knee injuries, the prevalence of OA continues to grow and is projected to rise further in the coming decades [3, 4, 5]. In the United States alone, OA affects 20 to 30 million individuals, resulting in an estimated annual economic loss of 60 billion US dollars [6]. A study of adults aged 20–30 years in Eastern Europe reported that 37.05% of participants exhibited at least one form of temporomandibular joint (TMJ) disorder, with 17.79% of affected individuals classified as having joint pain, arthritis, or degenerative joint disease, including temporomandibular joint osteoarthritis (TMJOA). Furthermore, the affected population included a considerably higher proportion of women than men [7]. Despite substantial clinical demand, OA pathogenesis remains incompletely understood, and effective disease-modifying OA therapies are still lacking [6, 8]. TMJOA is a multifaceted degenerative disorder characterized by progressive cartilage degeneration, chronic pain, and restricted TMJ function, substantially impairing patients’ quality of life [9].

The occurrence of OA is influenced by multiple factors, among which aging represents the most critical determinant in its pathogenesis. OA development is closely associated with the progressive accumulation of senescent cells within joint tissues. Evidence indicates that cartilage degradation and OA occurrence are linked to the senescence-associated secretory phenotype (SASP). Moreover, age-related mitochondrial dysfunction and consequent oxidative stress contribute to cellular senescence in joint tissues [10, 11, 12]. During the aging process, the accumulation of senescent cells across various tissues promotes chronic inflammation, thereby accelerating age-related functional decline [13].

Aging is driven by multiple intrinsic and extrinsic factors (Fig. 1), including replicative stress, telomere attrition, oxidative damage, metabolic dysfunction, cytokines, oncogene activation, and chemotherapeutic agents. These factors induce cellular damage and senescence in normal cells and, in certain contexts, in cancer cells [14]. This review examines the role of cellular senescence in OA pathogenesis and treatment, thereby establishing a foundation for developing novel therapeutic strategies for OA and TMJ arthritis.

**Fig. 1.** Molecular mechanisms of cellular senescence in OA. This diagram illustrates how aging-related signaling pathways contribute to OA by inducing oxidative stress and SASP. Mitochondrial dysfunction results in insufficient adenosine triphosphate production, reduced FOXO activity, activation of the Piezo1/Ca^2+^ channel in chondrocytes, and suppression of AMPK activity, collectively leading to increased ROS generation. The resulting oxidative stress promotes the release of matrix-degrading enzymes, including MMPs and ADAMTSs, thereby accelerating cartilage matrix degradation and culminating in OA onset. In addition, activation of the Piezo1/Ca^2+^ channel in chondrocytes by aberrant mechanical stress, upregulation of chronic inflammatory factors such as IL-1β and IL-6, and DNA damage-mediated activation of ATM and ATR converge on the NF-κB pathway, leading to SASP factor secretion. Ultimately, these processes drive OA progression through mechanisms analogous to those mediated by ROS. MMPs: matrix metalloproteinases; OA: osteoarthritis; GSH: glutathione; FOXO: Forkhead box; AMPK: Adenosine monophosphate-activated protein kinase; ROS: reactive oxygen species; SASP: senescence-associated secretory phenotype; IL: Interleukin; NF-κB: Nuclear Factor-kappa B Signaling Pathway; ATM: Ataxia-Telangiectasia Mutated; ATR: Ataxia-Telangiectasia and Rad3-Related; ↑: increased expression; ↓: decreased expression; ADAMTSs: A Disintegrin And Metalloproteinase with ThromboSpondin Motifs; ATP: Adenosine Triphosphate.

## 2. Cellular senescence

Cellular senescence was first proposed by Hayflick and Moorehead in 1961 as the irreversible loss of replicative potential in primary cells cultured *in vitro* [15]. This irreversible growth arrest arises in response to deleterious stimuli, including DNA damage, telomere attrition, mitochondrial dysfunction, and oncogenic stress, thereby preventing proliferation of potentially dysfunctional, transformed, or senescent cells [16]. Currently, cellular senescence denotes a state of irreversible cell cycle arrest accompanied by alterations in cell morphology, secretory profiles, and epigenetic regulation [17]. Senescence is a highly dynamic, multistep process in which cellular characteristics constantly evolve and diversify [18]. The prevailing definition of senescence requires the establishment of permanent cell cycle arrest. Accordingly, cells are not classified as senescent until permanent cell cycle arrest is achieved, even if other hallmark features, such as pro-inflammatory phenotypes, emerge earlier and depend on stable cell cycle arrest.

Cellular senescence, as a principal contributor to diseases, impairs long-term tissue regeneration and normal cellular function and is associated with sarcopenia, osteoporosis, OA, macular degeneration, pulmonary insufficiency, renal failure, and neurodegenerative disorders, including Alzheimer’s disease and Parkinson’s disease. Moreover, senescence plays a key role in the early stages of wound healing and embryogenesis [19, 20].

### 2.1 Telomere shortening

Telomere shortening is a defining mechanism of cellular senescence. According to the replicative senescence theory, in the absence of telomerase activity, telomeres at chromosome termini progressively shorten with each DNA replication cycle. Upon reaching a critical length, telomeres resemble DNA double-strand breaks (DSBs), activating DNA damage responses and inducing cell cycle arrest [14, 21]. Telomere attrition and consequent decline in proliferative capacity have been closely associated with numerous age-related diseases, with telomere length shortening being more progressively exacerbated in highly proliferative cells [22].

### 2.2 DNA damage

During cellular growth, various endogenous factors, including replicative stress, metabolic byproducts, telomere damage, reactive oxygen species (ROS), as well as exogenous factors such as ionizing radiation and chemical toxins, can induce genomic damage. This damage manifests as base mismatches or deletions, replication and transcription blocks, single-strand DNA breaks, and DSBs [23]. DNA damage typically arises from normal cellular processes, with DSBs representing the most severe form of DNA damage. Multiple cell cycle checkpoints respond to DNA damage, with several effector proteins shared across different checkpoints [24, 25]. The cross-interaction between DNA Damage Response (DDR) and the cell cycle facilitates DNA damage repair or induces senescence or apoptosis when repair fails [26, 27].

### 2.3 Mitochondrial dysfunction

Current research has demonstrated that cellular senescence is highly heterogeneous [28], with pronounced variability in senescence-associated changes among individual cell lineages. This heterogeneity is closely linked to stress responses, particularly oxidative stress mediated by ROS. Under physiological conditions, mitochondria constitute the primary source of ROS in cells [29]. The mammalian target of rapamycin complex 1 (mTORC1) integrates stress signals to regulate protein and lipid synthesis and autophagy, thereby influencing mitochondrial homeostasis [30]. Silencing mTOR via small interfering RNA has been demonstrated to reduce ROS and DNA damage foci in senescent fibroblasts [31, 32].

### 2.4 Senescence-associated β-galactosidase (SA-β-Gal)

SA-β-Gal is the most widely used biomarker of cellular senescence, defined by β-galactosidase activity detectable at pH 6.0 [33]. Analysis of peripheral blood from healthy individuals in their 20s and those over 60 revealed that SA-β-Gal activity in peripheral blood mononuclear cells increases with age. Moreover, aging is associated with a higher proportion of T lymphocytes, particularly Cluster of Differentiation 8-positive cells (CD8^+^ T cells), exhibiting elevated SA-β-Gal activity. These CD8^+^ T cells demonstrate features of telomere dysfunction-induced senescence and p16-mediated senescence [34].

### 2.5 Senescence-associated secretory phenotype (SASP)

Senescent cells exhibit extensive alterations in chromatin organization and gene expression, including the secretion of pro-inflammatory cytokines, chemokines, growth regulators, angiogenic factors, and matrix metalloproteinases (MMPs), collectively referred to as the SASP [11, 20]. Initially, SASP was considered to regulate a subset of senescence-related cytokines, including pro-inflammatory factors, immune modulators, and chemokines, such as interleukin (IL)-6 and IL-8 [35]. Subsequent research has demonstrated that SASP comprises hundreds of protein and non-protein signaling molecules, including coagulation factors, proteases, kinins, extracellular matrix (ECM) components, and damage-associated molecular patterns [20, 36, 37]. Knockout of H2A histone family member J (H2A.J) suppresses the expression of SASP inflammatory genes, whereas H2A.J overexpression increases their expression in proliferating cells. Consequently, H2A.J accumulation promotes senescent cell signaling to the immune system and may contribute to chronic inflammation and aging-related diseases [38]. Moreover, p21 has been identified as closely associated with limb cell senescence in mice [39], and directly participates in regulating the expression of specific SASP components [40].

### 2.6 Stem cell exhaustion

Stem cell exhaustion represents the cumulative result of various aging-related damages [41, 42]. Studies in aged mice demonstrate a decline in overall cell cycle activity of hematopoietic stem cells (HSCs) and an increase in endogenous DNA damage in wild-type stem cells. Accumulated DNA damage appears to be a primary driver of age-dependent stem cell decline [43, 44]. Investigation of the functional heterogeneity of microcephalin (MCPH1) in the nuclei and cytoplasm of mouse HSCs revealed that aging induces MCPH1 translocation from the cytosol to the nucleus, reducing its cytoplasmic pool, activating necroptosis, and impairing HSC function. Consequently, DNA damage-induced MCPH1 redistribution promotes HSC aging and may exert a more profound influence on aging and aging-related diseases [45].

## 3. Osteoarthritis (OA)

Arthritis is the most common degenerative joint disease, affecting single or multiple joints, including small joints, such as hand and TMJs, and large joints, such as knee and hip joints. OA is recognized as a disease of the entire joint, encompassing alterations in articular cartilage, subchondral bone, ligaments, joint capsules, and synovium, ultimately resulting in joint failure. Primary OA arises from a combination of risk factors, including knee misalignment, increased biomechanical joint load, genetic predisposition, and low-grade systemic inflammation, with aging and obesity representing the most significant contributors [46, 47].

### 3.1 Risk factors for OA

#### 3.1.1 Age

Among the various risk factors for OA, age is the most significant risk determinant of its onset and progression. The incidence of OA rises with advancing age, affecting the knee, hip, and hand joints [48]. An analysis of OA prevalence across age groups reported the following rates: 1.8% for individuals aged 40–49 years, 9.6% for 50–59, 14.7% for 60–69, 26.9% for 70–79, and 27.5% for those aged 80 and above. Although no significant change was observed with each 5-year age increment, prevalence increased markedly with each 10-year increment [49].

#### 3.1.2 Obesity

Obesity is a major contributor to hip and knee OA. Excess body weight imposes abnormal mechanical stress on the knee joints, exceeding physiological limits, which accelerates cartilage wear and tear, often damages joint ligaments, and ultimately leads to OA. Beyond weight-bearing joints, obesity also considerably affects the hand joints, which are non-weight-bearing; the incidence of hand OA is higher in obese individuals compared with those with normal body weight [50]. Adipocytes in obese individuals secrete cytokines, termed “adipokines”, including leptin, resistin, retinol, and adiponectin, which promote OA occurrence and progression [51].

#### 3.1.3 Gender

Epidemiological studies conducted between 2012 and 2016 report an overall OA prevalence of 14.6%. The prevalence of knee OA is higher in females (19.1%) than in males (10.9%), indicating a greater susceptibility among women. Corroborating evidence from the United Kingdom demonstrates that females face an elevated risk of developing knee OA compared with males, with a 5-year incidence of 17.6% in women aged 45–65 years [52]. Declining sex hormone levels in menopausal women may contribute to OA onset and progression [53].

### 3.2 OA pathogenesis

#### 3.2.1 Cartilage

Cartilage comprises chondrocytes embedded in an ECM. Chondrocytes maintain cartilage homeostasis by synthesizing an ECM enriched in type II collagen, proteoglycans, and associated macromolecules. Aging or excessive mechanical stress induces abnormal catabolic activity in chondrocytes. Aged chondrocytes exhibit reduced oxidative stress resistance and impaired autophagy, compromising cellular homeostasis and initiating OA [54]. Hou *et al*. [55] report that toll-like receptor 3 (TLR3) accelerates OA progression through two mechanisms: it activates the pro-inflammatory Nuclear Factor-kappa B Signaling Pathway (NF-κB pathway), promoting cartilage matrix degradation, and suppresses autophagy proteins, disrupting cellular homeostasis.

#### 3.2.2 ECM and pericellular matrix (PCM)

The earliest alteration in OA onset occurs in the matrix immediately surrounding chondrocytes, termed the PCM. This region, approximately 2 to 4 micrometers in thickness, is located within the narrow ECM adjacent to chondrocytes and is primarily composed of type VI collagen, fibronectin, and proteoglycans [56]. Rahmati *et al*. [57] have demonstrated that enhanced ECM catabolism is a central driver of OA onset and progression. Within the osteoarthritic inflammatory milieu, cellular metabolism shifts toward enhanced glycolysis to meet the increased energy demands of chondrocytes. Lactic acid, a key glycolytic metabolite, is elevated in synovial fluid from patients with OA and correlates with increased ROS production, upregulated MMP expression, and suppression of anabolic genes such as collagen II, ultimately leading to ECM degradation [58]. Fu *et al*. [59] reported that excessive ECM stiffening induces overactivation of mitochondrial autophagy, thereby accelerating cartilage senescence. Senescent chondrocytes subsequently release deleterious mediators that disrupt ECM synthesis and degradation, ultimately driving OA progression.

#### 3.2.3 Subchondral bone

Subchondral bone consists predominantly of mineralized type I collagen and, together with articular cartilage, forms the articular surface of the joint. Alterations in the structural and functional interactions between subchondral bone and articular cartilage constitute key drivers of OA development [60]. Subchondral bone comprises two distinct components: the subchondral bone plate and the subchondral trabeculae. Accumulating evidence links subchondral bone metabolism to OA pathogenesis, with stage-dependent variations across disease progression. During early-stage OA, thinning of the subchondral bone plate occurs alongside an increase in subchondral trabecular number. Conversely, during late-stage OA, the subchondral bone plate thickens, and the trabeculae exhibit increased density [61]. Wu *et al*. [62] reported that chondrocyte internalization of exosomes derived from OA-hardened subchondral osteoblasts induces catabolic gene expression and suppresses chondrocyte-specific marker levels. MicroRNA (miRNA)-210-5p, highly enriched in these exosomes, promotes catabolic gene expression in articular chondrocytes and reduces their oxygen consumption rate, thereby altering cellular bioenergetics during OA progression. Inhibition of miR-210-5p markedly attenuates these effects, highlighting the critical role of subchondral osteoblast-derived exosomes in OA progression. Guan *et al*. [63] identified that sympathetic innervation facilitates the transfer of miRNA-125 from osteoarthritic chondrocyte-derived exosomes, ultimately disrupting subchondral homeostasis and exacerbating cartilage degeneration in aged mice. These findings suggest that the sympathetic nervous system may regulate age-related OA, providing novel insights for disease management.

#### 3.2.4 Synovial membrane

The synovium is the connective tissue that surrounds joints, providing structural support, lubrication, and nutritional supply. Synovitis occurs at all stages of OA and is associated with synovial fibrosis in advanced disease. Fibroblast-like synoviocytes (FLSs) are central regulators of OA pathogenesis. FLS proliferation disrupts the balance between connective tissue synthesis and catabolism, promoting the release of inflammatory mediators and catabolic factors, thereby exacerbating synovial inflammation and contributing to cartilage degeneration and OA progression [64, 65].

## 4. Cellular senescence and OA

### 4.1 DNA damage

DNA damage can induce telomere dysfunction, leading to chromosomal instability and promoting the progression of cancerous lesions. In the absence of DNA damage, telomere dysfunction triggers cellular senescence, resulting in a stable and permanent cell cycle arrest [66]. Poonpet *et al*. [67] reported that leukocytes from patients with knee OA exhibit shorter relative telomere length than healthy controls. Rossiello *et al*. [68] observed that, in patients with hip OA, cells located closer to the hip joint exhibited reduced telomere length and increased expression of aging-associated markers compared with cells situated farther from the affected site. Senescence requires the regulation of at least two signaling pathways: the p16-dependent pathway and the p53-mediated DNA damage response, which is characterized by DNA DSBs [69]. Telomere dysfunction may be recognized as a DNA DSB, thereby activating the p53-induced senescence pathway [21, 70]. DNA damage typically activates the canonical p53–p21 axis, leading to early cell-cycle arrest (early senescence), whereas chronic or unresolved damage induces p16 upregulation. Upon activation, p53 undergoes transient phosphorylation at Ser15/20 to escape Mouse Double Minute 2-mediated protein degradation (MDM2-mediated degradation), promoting transcription of p21, a cyclin-dependent kinase inhibitor. Subsequently, p21 binds to Cyclin-Cyclin-Dependent Kinase 2/4 Complexes (cyclin-CDK2/4 complexes), blocking the G_1_→S phase transition and inducing reversible early cell-cycle arrest, serving as a “protective brake” that allows DNA repair. In osteoarthritic chondrocytes, p21 is often elevated during early disease stages [71], whereas p16 is predominantly upregulated in later stages [72]. Although p53 expression may not remain elevated, its activation initiates the senescence cascade.

### 4.2 Chronic inflammation

Chronic inflammation contributes to cellular senescence and constitutes a key factor in OA development [73]. Cytokines implicated in chondrocyte damage, including IL-6 and IL-1β, play a critical role in OA progression and are consistently present in affected joints [74]. These cytokines primarily signal through three pathways: (1) the Janus Kinase/Signal Transducer and Activator of Transcription Pathway (JAK/STAT pathway), (2) the Rat Sarcoma Virus Oncogene Homolog/Mitogen-Activated Protein Kinase Pathway (Ras/MAPK pathway), and (3) the Phosphatidylinositol 3-Kinase/Protein Kinase B Pathway (PI3K/Akt pathway) [75]. Through *in vivo* electrophysiological experiments, Mima *et al*. [76] reported that IL-6-induced JAK2/STAT3 activation promotes cartilage degeneration and arthritic pain during OA progression. Lan *et al*. [58] demonstrated that IL-1β stimulation progressively increases pan-glutamylation levels in chondrocytes. IL-1β-mediated activation of the MAPK pathway induces ECM degradation and chondrocyte apoptosis, thereby contributing to OA. Additionally, PI3K and Akt undergo rapid phosphorylation in response to IL-1β stimulation [77]. Li *et al*. [78] demonstrated that IL-1β significantly upregulates p-PI3K, p-Akt, and p-mTOR in chondrocytes, thereby activating the PI3K/Akt pathway and promoting OA development. Consequently, chronic inflammation represents a critical link between aging and OA, with aging-associated release of inflammatory mediators driving OA development.

### 4.3 Energy metabolism dysfunction

Adenosine monophosphate-activated protein kinase (AMPK) is a central regulator of cellular metabolism and energy homeostasis [79]. Multiple studies have demonstrated that AMPK mitigates age-related secretory phenotypes and extends lifespan [80, 81]. AMPK expression and robust phosphorylation are detectable in healthy joints. Evidence indicates that AMPK deacetylates p65 via Sirtuin 1 (SIRT1), promoting its proteasomal degradation and consequently inactivating NF-κB signaling, which reduces inflammation and joint damage. AMPK activation exerts a protective effect on articular cartilage [82]. Therefore, AMPK activation may decelerate aging and prevent OA progression.

### 4.4 Forkhead box (FOXO) transcription factors

The FOXO transcription factor family is a central regulator of genes involved in aging, lifespan, and responses to oxidative stress [83]. FOXO transcription factors confer protection against cellular and organismal aging; however, FOXO expression in cartilage declines with age and in OA. In chondrocytes, FOXO1 enhances TLR4-mediated inflammatory signaling. TLR4 activation triggers the downstream PI3K/Akt pathway, which inactivates FOXO1 via phosphorylation. Inactivated FOXO1 subsequently downregulates TLR4 expression, forming a negative feedback loop that limits excessive inflammatory amplification. Nevertheless, dysfunction or dysregulation of this negative feedback mechanism results in a persistent chronic inflammatory microenvironment within the joint, exacerbating intra-articular inflammation and promoting OA onset [84]. Consequently, FOXO functions as a protective factor, slowing age-related cartilage degeneration and reducing OA risk.

### 4.5 Oxidative stress

Oxidative stress refers to an imbalance between oxidants and antioxidants, resulting in excessive accumulation of ROS and consequent molecular damage, primarily regulated by thioredoxin and glutathione (GSH) [85]. This process induces senescence in human articular chondrocytes, while senescent cells demonstrate increased susceptibility to oxidative stress-mediated death due to impairment of the GSH oxidation system [86]. Elevated ROS levels induce low-grade inflammation in cartilage and synovium, stimulating the secretion of enzymes that degrade the ECM and accelerate cartilage degradation. Concurrently, oxidative stress suppresses the expression of cartilage-specific anabolic genes, such as aggrecan (*ACAN*) and collagen II alpha 1, thereby compromising chondrocyte function and joint structure integrity [87, 88]. Zhou *et al*. [89] reported that miRNA-34a-5p participates in ROS-induced oxidative stress responses and subsequently regulates inflammatory processes through the SIRT1/p53 signaling pathway, thereby influencing OA progression. In summary, excessive ROS production disrupts intracellular signaling, chondrocyte life cycle, and cartilage matrix metabolism. These alterations promote synovial inflammation and subchondral bone remodeling, further driving OA development [90].

### 4.6 Mitochondrial dysfunction

Mitochondria represent the principal source of ROS and play a critical role in chondrocyte function. Mitochondrial dysfunction increases ROS production while reducing adenosine triphosphate synthesis, thereby promoting MMP expression and ultimately inducing chondrocyte senescence and OA onset [91, 92]. Lin *et al*. [93] reported that SIRT4 knockdown in chondrocytes elevated ROS levels, disrupted mitochondrial morphology, and impaired mitochondrial membrane potential, indicating mitochondrial dysfunction. Conversely, lentiviral vector-mediated SIRT4 gene delivery successfully preserved articular cartilage integrity in a murine OA model. These findings suggest that SIRT4 knockdown promotes mitochondrial dysfunction, chondrocyte senescence, and OA progression. Additionally, Wang *et al*. [94] identified Peroxisome Proliferator-Activated Receptor Gamma Coactivator 1 Alpha (PGC-1α) as a key regulator of mitochondrial biogenesis with protective effects on cartilage. This factor delays OA onset and progression by modulating mitochondrial biogenesis, oxidative stress, mitochondrial autophagy, and chondrocyte DNA replication.

### 4.7 Mechanical stress

The generation and perception of mechanical forces are fundamental to organismal development and survival. Impaired mechanosensation contributes to the pathogenesis of multi-organ disorders, including skeletal muscle disease, cardiovascular disease, and renal failure [95]. As mechanosensitive transcriptional co-activators, Yes-associated Protein/Transcriptional co-activator with PDZ-binding motif (YAP/TAZ) regulate aging-related processes, including skin laxity, myocardial fibrosis, pulmonary function decline, and disruption of stem cell niche homeostasis [96, 97]. Piezo1, a mechanosensitive ion channel, facilitates the conversion of mechanical signals into cellular responses. In chondrocytes, aberrant Piezo1 activation is associated with aging, catabolic activity, and apoptosis [98]. Abnormal mechanical loading reduces chondrocyte activity [99], and Piezo1 expression is elevated in osteoarthritic chondrocytes. Consequently, Piezo1 contributes to OA development by altering chondrocyte mechanical properties and promoting catabolic processes [98]. Han *et al*. [100] have demonstrated that excessive mechanical stress induces OA by promoting ECM degradation through Piezo1-mediated activation of the PI3K/Akt/mTORC1 pathway in chondrocytes. Shao *et al*. [101] reported that excessive mechanical loading causes mitochondrial dysfunction, which subsequently triggers chondrocyte senescence and leads to OA onset. Accordingly, aberrant mechanical signals promote matrix degradation by activating PI3K/Akt/mTORC1 and integrin—FAK signaling pathways or inducing cellular senescence via mitochondrial dysfunction, thereby contributing to OA development.

## 5. Cellular senescence and TMJOA

OA is the most common degenerative condition among TMJ disorders and often manifests as limited mouth opening, joint pain, and joint noises, leading to substantial impairment of patient quality of life. In contrast to articular cartilage, which consists primarily of hyaline cartilage, mandibular condylar cartilage is predominantly fibrocartilaginous [102]. These structural differences underlie distinct mechanisms of cellular aging in TMJOA and OA (Table 1, Ref. [102, 103, 104, 105, 106, 107, 108]). Moreover, subchondral bone thinning represents a common early pathological feature of OA [109], whereas TMJOA is characterized by concurrent condylar cartilage degeneration, calcification, and osteophyte formation [110]. Abnormal mechanical loading constitutes a major risk factor for TMJOA. Mechanical overload induces cellular senescence *in vitro*, and defects in YAP mediate alterations in mechanical signaling that promote senescence. Senescent cells subsequently accumulate in mechanically induced TMJOA lesions [111]. Furthermore, Cai *et al*. [112] demonstrated, through combined *in vivo* and *in vitro* experiments, that mechanical stress contributes to TMJOA progression via the Wnt/β-catenin signaling pathway. Chondrocyte ferroptosis also plays a critical role in TMJOA pathogenesis by disrupting mitochondrial structure and function and upregulating transferrin receptor 1, thereby increasing ferrous ion influx into chondrocytes, promoting ferroptosis, impairing chondrocyte function, and exacerbating condylar cartilage degeneration [113, 114].

**Table 1.** Comparison of OA and TMJOA.

Feature	OA	TMJOA
Cartilage type [102, 103]	Hyaline cartilage	Fibrocartilage
Main cell types [105]	Chondrocytes	Fibrocartilage cells and progenitor/stem cells
Primary collagen type [106]	Type II collagen	Type I collagen
Key aging focus [107]	Chondrocyte functional failure and ECM degradation	Progenitor cell pool depletion and coordinated senescence of multiple cell types
ECM aging hallmarks [106]	Disruption of the type II collagen network and loss of ACAN	Disorganization of type I collagen fibril bundles and generation of specific degradation fragments
Mechanical transduction [104]	Primarily responds to compressive stress	Responds to complex stresses (especially shear) and potentially more sensitive
Subchondral bone remodeling abnormalities [108]	Present but relatively slow	Exceptionally active and critical (observable within one week)

*OA: Osteoarthritis; TMJOA: Temporomandibular joint OA; ECM: extracellular matrix; ACAN: aggrecan.*

TMJ is a sliding-hinge composite joint whose integrity depends on the normal positioning and smooth gliding of the articular disc. Anterior displacement of the articular disc induces an immediate and pronounced change in the joint’s mechanical environment [104], generating abnormal shear forces and pressure that directly impact the fibrocartilage and subchondral bone of the condylar head. These mechanical changes rapidly initiate signal transduction pathways, resulting in cellular stress, inflammation, and accelerated aging. The knee is a weight-bearing hinge joint, and its aging primarily reflects cumulative “wear and tear” and “fatigue”. Prolonged weight-bearing stress gradually degrades the cartilage matrix, disrupting its proteoglycan content and collagen network.

The rate of repair cannot match the rate of degradation, reflecting a chronic and progressive process of mechanical inflammation in TMJ. This inflammation frequently progresses “from the outside in”, indicating that abnormal mechanical stress or local trauma initially disrupts the joint environment, subsequently triggering a cascade of local inflammatory responses. In contrast, knee joint inflammation frequently follows an “inside-out” process. A systemic state of “inflammatory aging” establishes a persistent pro-inflammatory milieu, increasing vulnerability of articular cartilage and its susceptibility to mechanical stress-induced damage. Table 1 summarizes the distinctions between OA and TMJOA.

## 6. Therapies

Two categories of SASP-modulating drugs have been investigated for treating OA: senolytics and senomorphics [115]. Senolytic agents alleviate age-related diseases by selectively inducing apoptosis in senescent cells [116]. In contrast, senomorphic therapies modulate senescent cell behavior by regulating cellular function and suppressing SASP secretion without inducing apoptosis, thereby addressing senescence-associated cellular dysfunction [117]. Dasatinib and quercetin can eliminate senescent cells from bone and cartilage tissues [118, 119]. Chondrocyte homeostasis is associated with peroxisome proliferator-activated receptor alpha (PPARα). Fenofibrate, a PPARα agonist, decreases senescent cell accumulation through apoptosis induction, prevents cartilage degeneration, mitigates senescence and inflammation, and enhances autophagy in chondrocytes of patients with OA, representing a promising intervention for age-related cartilage degeneration and OA [120]. Additionally, Arra *et al*. [121] demonstrated that inhibition of Inhibitor of κB-zeta (IκB-ζ) reduces SASP secretion by regulating the NF-κB–IκB-ζ axis, thereby controlling joint inflammation. Table 2 (Ref. [63, 121, 122, 123, 124, 125, 126, 127, 128, 129, 130, 131, 132, 133, 134, 135]) summarizes the cellular senescence mechanisms of senolytics and senomorphics, highlighting their therapeutic potential in OA management.

**Table 2.** Therapeutic mechanisms of senolytics and senomorphics in OA.

Class	Compound	Mechanism of action
Senolytics		
	Dasatinib and quercetin [126]	Selectively eliminate senescent cells, reduce inflammation, and mitigate ECM degradation
	ABT-263 [127] ABT-737 [127]	Inhibits BCL-2 family proteins, selectively promoting senescent cell clearance
	ESC-sEVs [123]	Regulates FOXO1A-autophagy axis and rejuvenates aged chondrocytes
	ASC-EVs [124]	Reduces senescence markers and inflammation
	miR-325-3p [125]	Modulates p53/p21 signaling and alleviates facet joint degeneration
	miR-125 [63]	Activates β1-adrenergic receptor signaling and promotes osteoblast differentiation in subchondral bone
	Fisetin [128]	Activates SIRT6 and reduces ECM degradation
	Fenofibrate [122]	Increases PPARα expression, decreases chondrocyte senescence, and enhances autophagic flux
Senomorphics		
	Procyanidin B2 [129]	Modulates Nrf2/BAX/BCL-2, reduces SASP, and prevents cartilage degradation
	HQC [130]	Inhibits STAG1/TP53/P21, suppresses SASP, and reduces chondrocyte senescence
	IκB-ζ inhibitor [121] Dendrobine [131]	Regulates NF-κB and suppresses SASP
	Rhoifolin [132]	Modulates Nrf2/NF-κB and reduces SASP factors
	Pterostilbene [133]	Inhibits PI3K/Akt/NF-κB, reduces SASP, and increases collagen type II
	Isoquercetin [134]	Inhibits NOX4 and suppresses SASP
	Rapamycin [135]	Reprograms senescent cells, reduces SASP factors, and delays cartilage degeneration

*ESC-sEVs: embryonic stem cell–derived small extracellular vesicles; ASC-EVs: adipose-derived stem cell–derived extracellular vesicles; HQC: Huangqin Qingre Chubi Capsules; IκB-ζ inhibitor: Inhibitor of κB-zeta; ECM: extracellular matrix; SIRT6: Sirtuin6; NOX4: NADPH oxidase-4; FOXO1A: Forkhead box; PPARα: peroxisome proliferatoractivated receptor alpha; SASP: Senescence-associated secretory phenotype; Nrf2: Nuclear factor-erythroid 2 related factor 2; BCL-2: B-cell lymphoma 2; BAX: BCL-2-Associated X; NF-κB: nuclear factor kappa-B; PI3K: Phosphoinositide 3-kinase; Akt: Protein Kinase B signaling pathway; STAG1: Stromal Antigen 1.*

Stem cell depletion represents a key hallmark of human aging. The age-related decline in the function of intra-articular mesenchymal stem cells (MSCs) has been identified as a major contributor to OA onset [120]. Yu *et al*. [136] reported that downregulation of YAP-FOXO1 pathway accelerates MSC senescence. Concurrently, upregulation of senescence markers, including p16 and p21, further impairs the regenerative capacity of these cells. Beyond conventional stem cell transplantation, exosome-based therapies, embryonic stem cell–derived small extracellular vesicles (ESC-sEVs), adipose-derived stem cell–derived extracellular vesicles (ASC-EVs), and miRNAs such as miR-325-3p and miR-125, represent novel strategies for OA treatment. The therapeutic efficacy of these interventions is mediated through multiple mechanisms, including anti-inflammatory effects, regulation of cellular metabolism, and promotion of cartilage regeneration [63, 123, 124, 125].

Extracellular vesicles (EVs) secreted by MSCs effectively enhance the proliferation, differentiation, survival, and migration of stem and progenitor cells in dental and mandibular tissues while promoting vascular and neural regeneration. Integrating these technologies may enable more precise therapeutic strategies and improve patient outcomes, offering new prospects for the management of OA and TMJ arthritis [19].

## 7. Limitations

Several limitations exist in the current literature that warrant further consideration. First, anti-senescence therapies, including senolytic and senomorphic drugs, demonstrate potential in improving OA; however, their clinical efficacy and long-term outcomes remain insufficiently characterized. Second, using SASP-targeting drugs for OA presents specific challenges. While effective in eliminating senescent cells, senolytics lack precise targeting and may inadvertently damage subpopulations of these cells that are essential for tissue repair, thereby compromising the repair capacity of joint tissues. Third, senomorphics, although capable of suppressing pro-inflammatory factors, such as IL-6 and tumor necrosis factor-alpha, may also inhibit beneficial inflammatory signals necessary for tissue repair, potentially delaying the healing process. Fourth, modulation of single SASP-regulatory pathways, including NF-κB and MAPK, can activate other signaling pathways. For instance, rapamycin inhibits the mTOR pathway to reduce inflammation, but may simultaneously enhance the expression of fibrosis-related factors, including transforming growth factor-beta, thereby increasing joint stiffness. Fifth, although SASP-targeting drugs are beneficial in OA treatment, they may induce off-target effects in other organs. Agents such as SIRT activators and mTOR inhibitors can disrupt systemic metabolic pathways and, with prolonged use, may cause blood glucose fluctuations, dyslipidemia, or impair hepatic and renal function. Consequently, selecting appropriate SASP-targeting drugs based on distinct pathogenesis mechanisms remains a critical challenge and a primary focus of ongoing clinical research.

This review examines the differences between OA and TMJ arthritis in terms of tissue structural characteristics and cellular aging-related pathological mechanisms. Nevertheless, the heterogeneity of cellular aging regulatory pathways, key biomarkers, and microenvironmental interactions across other degenerative joint diseases, including rheumatoid arthritis, ankylosing spondylitis, and psoriatic arthritis, has not been systematically analyzed. Consequently, future research should investigate these diseases in greater detail.

## 8. Conclusions

Cellular senescence disrupts joint microenvironment homeostasis by impairing the function of multiple cell types, including chondrocytes, synovial cells, and osteoblasts. This process is recognized as a central pathological driver of OA, including TMJOA. These effects are mediated through SASP secretion, mitochondrial dysfunction, and the amplification of chronic inflammatory responses. Future research should prioritize the identification of specific biomarkers and regulatory pathways that govern dominant aging subtypes, including replicative and stress-induced senescence, across distinct joint tissues, such as condylar cartilage, articular disc, synovium, and subchondral bone. Research should also aim to elucidate the diffusion patterns of SASP factors within heterogeneous joint microenvironments and the mechanisms underlying their interaction with target cells. This approach will enable the identification of precise molecular targets for targeted therapeutic interventions. To accelerate clinical translation, standardized diagnostic protocols for aging classification must be established rapidly. In parallel, the advancement of small-scale clinical trials evaluating disease-modifying drugs, including senolytics, is essential. Additionally, long-term mechanical performance and biocompatibility of three-dimensional-printed prostheses require optimization [19]. Three-dimensional printing technology facilitates the fabrication of patient-specific joint implants tailored to individual anatomical characteristics, thereby improving the joint replacement outcomes. Zhan *et al*. [137] developed a rat OA model using anterior cruciate ligament transection and destabilization of the medial meniscus. Four weeks post-surgery, intra-articular injections of phosphate-buffered saline, Control Extracellular Vesicles (CON-EV), or IL-6-EV demonstrated that IL-6-EV markedly reduced cartilage damage. Mechanistic analyses confirmed that IL-6-EV enhances mitochondrial oxidative phosphorylation in macrophages by regulating the Mitochondrial NADH Dehydrogenase Subunit 3/NADH-Coenzyme Q Oxidoreductase Axis (mt-ND3/NADH-CoQ axis), thereby modulating macrophage polarization and alleviating OA. These findings underscore the therapeutic superiority of MSC-EVs following OA-specific microenvironment pretreatment and provide novel insights into their clinical application. Ultimately, dismantling barriers between basic research and clinical implementation and achieving precision, personalized prevention and treatment of OA requires multidisciplinary collaboration across orthopaedics, materials science, and medical imaging.
